# First-in-human to proof-of-concept: why experimental medicine studies remain essential in human physiology and drug development

**DOI:** 10.1007/s00424-026-03184-x

**Published:** 2026-06-04

**Authors:** Robert J. Unwin, Benjamin Challis, Carsten Wagner, Giovambattista Capasso, Stefan Carlsson

**Affiliations:** 1 UCL Centre for Kidney and Bladder Health, Division of Medicine, University College London (UCL), London, UK; 2 Translational Science and Clinical Development, Early CVRM, AstraZeneca BioPharmaceuticals R&D, Cambridge, UK; 3 Institute of Physiology and Zurich Kidney Center, University of Zurich, Zurich, Switzerland; 4 Biomedical and Molecular Genetics Research Institute, Ariano Irpino, AV Italy; 5 Caliditas Therapeutics AB, Stockholm, Sweden

**Keywords:** Experimental medicine, Chronic kidney disease (CKD), N-of-1 trials, Drug development, Mechanistic studies, Translational physiology

## Abstract

Experimental medicine studies, small, mechanistically focused investigations, have historically driven key discoveries in human physiology and pharmacology. Despite their foundational role, these studies are increasingly marginalised in today’s drug development environment due to economic pressures, regulatory conservatism, and an overemphasis on statistical endpoints from large-scale trials. This article traces the historical roots and enduring value of experimental medicine, distinguishes it from current early phase drug development studies, and explores the structural forces behind its decline. N-of-1 trials are discussed as a systematic extension of these principles, offering precision insights at the individual level. We apply this discussion to chronic kidney disease (CKD), a field where slow progression and heterogeneous pathophysiology make early mechanistic studies especially valuable. We argue that bypassing such studies in favour of speed represents a strategic gamble that may misdirect costly late-phase trials. Integrating mechanistic insights with statistical power is not superfluous, but essential, particularly in complex diseases like CKD where understanding why and how interventions work may matter as much as whether they do. We acknowledge that achieving this vision necessitates overcoming significant structural, economic, and cultural barriers within the current drug development environment; however, the costs of inaction, manifest as trial failures, patient harm, and missed therapeutic opportunities, are potentially much greater.

The experimental method is not the statistical method; the latter is a method of observation, which can be very useful for certain problems, but which cannot replace experimentation.

Claude Bernard, An Introduction to the Study of Experimental Medicine (1865) [3] .

## Introduction: the paradox of scale in medical discovery

An over-reliance on statistical power often comes at the expense of clinical insight [31]. While large randomised controlled trials (RCTs) are the statistical gold standard, this elevation has inadvertently pushed aside the value of small studies focused on carefully observed volunteers, often dismissed as anecdotal. Yet the history of medical discovery tells a very different story. Some of our most transformative insights emerged not from huge trials but from a few individuals studied with extraordinary precision: our understanding of insulin sensitivity came from clamp studies involving fewer than ten participants; the role of nitric oxide in vascular tone emerged from invasive physiological studies in small cohorts; and the bacterial cause of peptic ulcers was demonstrated when Barry Marshall drank a beaker of *Helicobacter pylori*, a study with *N* = 1. It is ironic that we demand large studies to prove what works, yet we often discovered how things work through small ones.

Leon Fine posed this very question a decade ago in the context of ‘big data’ [12] and the subsequent rise of artificial intelligence and machine learning in drug discovery makes it even more pressing. Today’s large multinational pharmaceutical companies are facing patent expiries, generic competition, and shareholder demands, and are under growing pressure to move fast and think big. In this environment, experimental medicine studies are increasingly viewed as non-registrational luxuries that industry cannot easily afford. This marginalisation comes at a cost. In complex diseases like chronic kidney disease (CKD), where pathophysiology involves intricate interactions between glomerular injury, tubular dysfunction, fibrosis, inflammation, and cardiovascular comorbidity, proceeding to large trials without a proper mechanistic understanding is like navigating without a compass. Large trials remain indispensable for demonstrating efficacy and detecting rare adverse events across heterogeneous populations; however, early mechanistic studies can reveal unexpected off-target effects and unanticipated physiological disturbances, allowing earlier identification of safety risks. Consistent with analyses showing that 17–35% of Phase 3 failures are attributable to safety rather than efficacy (50–57% of failures), experimental medicine should be viewed as a complementary approach that facilitates mechanism-based risk detection before widespread patient exposure [17].

In the era of multi-omics and systems biology, mechanistically focused studies in well-characterised, small cohorts can reveal biological signals that get lost in the noise of large, heterogeneous clinical trials. However, the registration of these experimental studies shows a significant shift in funding and design. Newly registered NIH-funded trials fell from 1,376 in 2006 to 1,048 in 2014, a decline of approximately 24% [10]. Completed NIH-sponsored Phase 3 to 4 trials, the large-scale efficacy studies often contrasted with mechanistic work, dropped most sharply, from 281 in the 2000–2004 period to just 28 by late 2019. Meanwhile, although industry-funded trials increased by 42.9% between 2006 and 2014, they represented only 22.2% of all trials started in 2020. The dominant force in the trial landscape is now the ‘Other’ funder category (including universities and hospitals), which sponsored 76.6% of trials in 2020, many of which fall into the increasing ‘non-drug research’ or ‘alternative design’ trials for non-FDA-defined interventions [15].

## Historical foundations: when scientists went first

Before ethics committees, institutional review boards, and regulatory frameworks, medical research operated on a different principle: if you believed in your hypothesis strongly enough, you tested it on yourself. Lawrence K. Altman’s seminal work, *Who Goes First?* [26], chronicles this era of self-experimentation, acts that were often reckless, occasionally fatal, but sometimes revolutionary. In 1929, Werner Forssmann catheterised his own heart, threading a urethral catheter from a vein in his arm into his right atrium and documenting it by X-ray [13]; this act of defiance earned him dismissal by his employer and, eventually, the Nobel Prize in 1956. Barry Marshall’s 1984 self-experimentation with *Helicobacter pylori* challenged decades of dogma about peptic ulcer disease [27], fundamentally transforming treatment and earning him a Nobel Prize in 2005. These are not relics of a bygone age but illustrations of an enduring principle: sometimes the most important insights come from a handful of carefully observed individuals rather than large population studies.

## Small studies driving large understanding: physiology and pharmacology

As research ethics evolved and self-experimentation gave way to structured protocols, experimental medicine became a systematic method of discovery. These investigations do not aim to prove that treatments ‘work’ in the clinical sense. Instead, they answer more fundamental questions: What does this intervention do to human biology? How does it do it? In whom does it work, and why? Table 1 summarises landmark examples across disciplines. The pattern is consistent: deep observation in small cohorts, often fewer than twenty participants, generating principles that apply broadly and transforming clinical practice. The fundamental approach remains unchanged: study a few patients intensively to understand biological principles. Modern experimental medicine has embraced sophisticated technologies while maintaining this focus on mechanisms, from microdialysis catheters sampling interstitial fluid in real-time, to stable isotope tracers following metabolic pathways with atomic precision, to continuous sampling methods revealing dynamic hormonal patterns undetectable by conventional single-timepoint measurement [24] .

**Table 1:** Landmark experimental medicine studies that transformed clinical practice. Each involved fewer than 20 participants and often far fewer

Domain	Study / Discovery	*N*	Key insight	Therapeutic impact
Diabetes/Metabolism	Hyperinsulinaemic–euglycaemic clamp (DeFronzo 1979) [7]	< 10	Precise quantification of insulin sensitivity and β-cell function	Mechanistic foundation for metformin, thiazolidinediones, GLP-1 agonists
Cardiovascular	Endothelium-derived relaxing factor/NO (Furchgott 1980; Palmer 1987) [14, 32]	~ 20	Endothelial cells release NO to relax vascular smooth muscle	Reshaped vascular biology; nitrate and related therapies
Neuropsychiatry	PET receptor occupancy studies (Farde 1992) [11]	< 15	60–80% D2 receptor occupancy required for antipsychotic efficacy; higher occupancy causes extrapyramidal effects	Dosing strategies for all antipsychotic drug classes
Gastroenterology	H. pylori self-experiment (Marshall 1984) [27]	1	Bacterial cause of peptic ulcers	Triple therapy; Nobel Prize 2005
Renal physiology	Renal clearance and RAAS mapping (Ambard 1912; Smith 1951) [19, 22]	< 20	Quantitative assessment of kidney function; renin–angiotensin regulation	ACE inhibitors, ARBs — cornerstones of cardiovascular and renal protection
Endocrinology	Continuous hormone sampling (Lightman 2008) [24]	< 10	Ultradian and circadian cortisol pulsatility invisible to spot sampling	Revised understanding of HPA axis dynamics; chronotherapy implications
Cardiology	Self-catheterisation of the right heart (Forssmann 1929) [13]	1	Right heart accessible via peripheral vein	Foundation of interventional cardiology; Nobel Prize 1956

## Defining the terms: experimental medicine vs. early phases of drug development

It is crucial to distinguish experimental medicine by its goals and approach rather than by regulatory phase designation. Pharmaceutical early development terminology — Phase 1b, Phase 2a, Phase 2b — has evolved inconsistently across companies and regulatory jurisdictions. What one company calls Phase 1b, another might designate Phase 2a. Increasingly, industry uses ‘Phase 2a’ to describe exploratory studies seeking statistically significant changes in surrogate biomarkers, which may or may not include dose-ranging.

The key distinction is not nomenclature but intent and methodology. Experimental medicine, whether labelled Phase 1b, Phase 2a, or conducted in academia outside formal phase designations, is characterised by: a hypothesis-driven mechanistic focus on understanding ‘why’ and ‘how’ an intervention works, not merely ‘whether’ it demonstrates preliminary efficacy; deep physiological interrogation through intensive measurement of biological mechanisms (e.g., renal haemodynamics, tissue oxygenation, metabolic flux, receptor occupancy, pathway activation) rather than reliance on routine laboratory endpoints; individual-level biological insight from in-depth data per participant, facilitating understanding of response heterogeneity; and flexibility for real-time hypothesis refinement based on emerging biological signals.

In contrast, traditional Phase 2 studies primarily seek to demonstrate preliminary efficacy, establish dose-response relationships, and identify common adverse events, with mechanistic assessments being secondary or absent. Some contemporary Phase 2a programmes blend these approaches, incorporating mechanistic endpoints alongside efficacy assessments, a trend we welcome and would advocate expanding. The critical point is that proceeding to large outcome trials without mechanistic understanding, regardless of phase designation, represents the strategic risk this article tries to address.

## Strengths, limitations, and mitigation strategies

Small mechanistic studies offer distinctive advantages in experimental medicine. Their precision allows targeting of specific biological pathways, with detailed assessment of tissue-level effects and biomarker dynamics that traditional clinical endpoints may miss. Intensive monitoring of fewer participants allows deeper understanding of individual responses, while tighter control of confounding variables and sophisticated measurement equipment may provide a level of accuracy often impractical in large, multi-centre trials.

Experimental medicine can extend safety evaluation beyond conventional Phase 1 toxicology by elucidating human mechanisms of action under controlled conditions. The mechanistic basis of euglycaemic ketoacidosis with SGLT2 inhibitors, arising from effects on insulin, glucagon, and hepatic ketogenesis in those with underlying beta-cell failure, illustrates how early mechanistic insights can support risk mitigation and patient selection before extensive safety datasets are available [34]. For personalised treatment, N-of-1 trials can identify what works for whom and why. Direct measurement of physiological readouts such as GFR, tubular reabsorption, and endothelial function yields information of high value that complements traditional clinical outcomes.

However, temporal considerations are critical. While renal haemodynamics and acute biomarker responses can be evaluated over days to weeks, anti-fibrotic effects typically require 6–12 months or longer, even with sensitive biomarkers or advanced imaging [21]. This temporal heterogeneity necessitates tiered study strategies, with short-term proof-of-mechanism studies complemented by longer, resource-intensive evaluations for fibrosis, a challenge compounded by the lack of validated non-invasive fibrosis biomarkers with clinically practical response times.

Critics raise valid concerns (see Table 2). Statistical weakness poses the greatest challenge, with high risks of Type I (false positive) and Type II (false negative) errors compromising generalisability. Economic trade-offs cannot be ignored: the cost per patient in intensive mechanistic studies can be substantial, and stringent eligibility criteria increase screening failure rates, prolong recruitment, and inflate costs. Regulatory limitations mean that findings, however mechanistically compelling, may not satisfy approval standards. Selection bias in small, homogeneous cohorts may also underrepresent real-world diversity.

We try to address these concerns in Table 2, which pairs each limitation with proposed mitigation strategies. Overarching principles include greater standardisation in design through pre-specified endpoints, transparent protocols, centralised analysis, and data sharing. Well-designed mechanistic studies with physiological endpoints provide precision rather than statistical power, which are complementary to, and not a substitute for, large trials. We acknowledge that requirements for pre-specified endpoints may conflict with early-phase demands for flexibility; however, reliance on exploratory *post hoc* analyses has contributed to costly late-stage failures, outweighing the time required for more systematic early-phase study design.

**Table 2:** Limitations of small mechanistic studies and proposed mitigation strategies

Limitation	Nature of concern	Mitigation strategy
Statistical power	High risk of Type I and Type II errors; findings may not generalise to broader populations	Bayesian methods and mechanistic modelling; pre-specified endpoints; physiological precision can compensate for sample size
Cost per patient	Intensive monitoring is expensive; stringent eligibility criteria increase screening failures	Embed within existing clinical research infrastructure; shared facilities via NIHR/CTSA networks; $2–5 M vs. $100–300 M for Phase 3
Regulatory acceptance	Mechanistic data rarely substitutes for RCT endpoints; only 8 biomarkers qualified via FDA programme to date [5]	Advocate for expanded biomarker qualification; use mechanistic data for dose selection, stratification, and risk detection rather than as primary approval evidence
Selection bias	Homogeneous cohorts underrepresent real-world diversity in age, comorbidities, and genetic backgrounds	Federated data platforms (e.g. NIDDK Central Repository) for pooling across sites; series of studies exploring heterogeneity in subgroups
Temporal constraints	Short-term readouts miss delayed effects (e.g., anti-fibrotic therapies require 6–12 months) [21]	Tiered study design: short-term proof-of-mechanism complemented by longer biomarker evaluations; development of validated non-invasive fibrosis markers
Reproducibility	Exploratory *post hoc* analyses often yield non-reproducible signals	Pre-specified endpoints; standardised protocols for GFR, haemodynamics, metabolic flux; transparent data sharing; synthetic/shared control arms from registries [30]

## The pharmaceutical industry’s dilemma

**Fig. 1:**
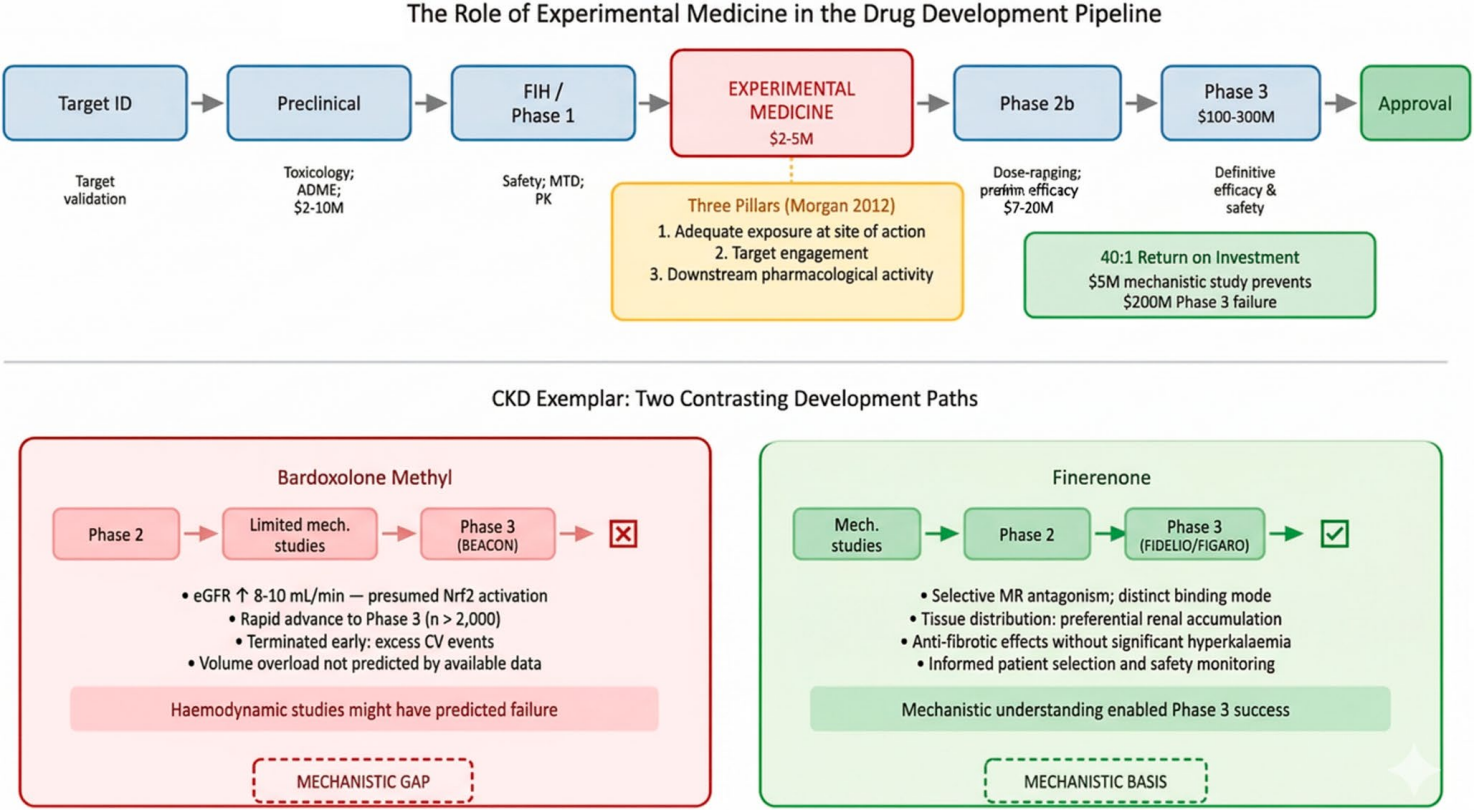
The role of experimental medicine in the drug development pipeline. *Upper panel*: development stages with associated costs and the questions each can address. The experimental medicine stage (highlighted) answers mechanistic questions at a fraction of Phase 3 costs. The ‘three pillars’ framework (Morgan 2012) identifies the conditions for successful development. *Lower panel*: contrasting development paths for bardoxolone methyl (limited mechanistic studies; Phase 3 failure) and finerenone (extensive mechanistic work; Phase 3 success) in CKD

The average cost of bringing a new drug to market in 2013 exceeded $2.6 billion [8], with timelines often stretching beyond a decade. In this high-pressure environment, experimental medicine studies present a paradox: they offer valuable insights but consume time and resources without directly advancing regulatory submissions (Fig. 1).

The economics of clinical development are increasingly challenging. While the median direct cost of a single pivotal efficacy trial is approximately $19.0 million, complex studies, such as those for cardiovascular indications, can average $157.2 million or reach as high as $346.8 million [28]. By contrast, intensive mechanistic studies in well-characterised cohorts offer a cost-effective alternative. For exploratory biomarker discovery, small cohorts of 6–10 participants can separate biological signals from variability; when embedded in research infrastructure, these studies can often be conducted for a fraction of the cost of large-scale trials. Yet facing a binary choice, companies often opt for larger trials, reasoning they can explore mechanisms later. However, with an industry-wide Phase 3 to approval success rate of only 59.0% (and a likelihood of approval from Phase 1 of just 13.8%), this calculation can be risky [40]. Analyses of drug attrition indicate that 65% of Phase II failures are due to insufficient efficacy [6]. Critically, a Pfizer analysis found that in 43% of Phase II failures, it was impossible to even determine whether the drug mechanism had been adequately tested [29]. To mitigate this, a landmark framework proposed the ‘Three Pillars of survival’ demonstrating target exposure, target binding, and pharmacological activity [29], while AstraZeneca’s ‘5R framework’ emphasises the ‘right tissue’ and ‘right patient’ to ensure mechanistic hypotheses are rigorously pressure-tested before investing in high-cost pivotal research [6]. Potentially, a $5 million mechanistic study that prevents a $200 million Phase 3 failure could represent a forty-fold return on investment.

## Forces driving decline

Several interconnected forces, regulatory, economic, organisational, and cultural, all conspire against experimental medicine in pharmaceutical development (see Table 3). These barriers are mutually reinforcing: regulatory conservatism reduces perceived value, which diminishes investment, which erodes expertise, which further reduces the quality and persuasiveness of mechanistic studies. Breaking this cycle means addressing multiple barriers simultaneously rather than relying on any single reform.

**Table 3:** Structural forces driving the decline of experimental medicine in drug development

Force	Mechanism	Consequence
Regulatory conservatism	Agencies acknowledge mechanistic data but rarely allow it to substitute for traditional endpoints; biomarker qualification progress is slow	Experimental medicine perceived as ‘nice to have’ rather than essential to development programmes
Ethical and societal barriers	Enhanced oversight introduces procedural complexity for small studies; heightened risk aversion and societal scepticism constrain exploratory physiological research	Recruitment difficulties and prolonged approval timelines for mechanistic investigations
Misaligned incentives	Project leaders rewarded for speed of compound advancement; investors prioritise enrolment numbers and timelines over biological insight	Systematic pressure to skip experimental medicine work even when scientifically justified
Safety underappreciation	Phase 1 defines MTD from acute toxicity but lacks resolution to anticipate chronic or population-specific toxicities; safety accounts for ~ 30% of clinical attrition [36]	Preventable late-stage safety failures that experimental medicine could have foreseen
Organisational silos	Early and late development teams often operate separately, sometimes on different continents; insights from mechanistic studies do not inform Phase 3 design	Mechanistic knowledge lost in translation between development stages
Loss of expertise	Downsizing or outsourcing of clinical pharmacology functions; erosion of institutional knowledge for sophisticated physiological investigation [2, 39]	Inability to design, conduct, or interpret experimental medicine studies even when resources are available

## CKD as an exemplar: where mechanism matters most

CKD exemplifies the risks of omitting mechanistic insight in trial design. The disease affects 850 million people globally [20], progresses silently over years or decades, and involves a complex interplay of haemodynamic, metabolic, inflammatory, and fibrotic pathways. Traditional large trials face enormous challenges: endpoints like doubling of serum creatinine or progression to dialysis take years to accrue, patient populations are heterogeneous, and treatment effects are often modest. In this context, experimental medicine studies can provide essential scientific value.

Consider two contrasting development paths (Fig. 1, lower panel). Bardoxolone methyl showed impressive eGFR improvements in Phase 2, increasing GFR by 8–10 mL/min/1.73m^2^ in diabetic kidney disease patients. The mechanism was presumed to involve Nrf2 activation and reduced oxidative stress. Enthusiasm was high, and the drug advanced rapidly to Phase 3 (BEACON) involving over 2,000 patients. But the trial was terminated early due to excess cardiovascular events [41]. Subsequent analysis suggested the drug might have caused volume overload and heart failure, effects that detailed haemodynamic studies might have predicted [4]. The failure cost hundreds of millions of dollars and, more importantly, exposed patients to harm.

Finerenone, in contrast, was supported by extensive mechanistic work before large trials. Experimental medicine studies demonstrated selective mineralocorticoid receptor antagonism with a distinct binding mode compared with spironolactone. Tissue distribution studies showed preferential kidney *versus* heart accumulation. Mechanistic studies in relatively small cohorts demonstrated anti-fibrotic effects without the more significant hyperkalaemia risk that had plagued earlier agents. This understanding informed patient selection and safety monitoring in large Phase 3 trials (FIDELIO-DKD and FIGARO-DKD), both of which succeeded [1, 33], leading to approval for diabetic kidney disease.

The difference is understanding not just whether a drug affects kidney function, but how it does so. Bardoxolone’s eGFR increase might have reflected haemodynamic changes rather than true nephroprotection, a distinction that invasive renal haemodynamic studies could have clarified. We recognise that this comparison is imperfect, reflecting the substantially greater resources available for finerenone’s development. Rather than suggesting smaller companies should undertake unaffordable programmes, this contrast highlights the need for shared infrastructure, academic partnerships, precompetitive consortia, and physiologically informed contract research organisations, to make systematic mechanistic evaluation possible irrespective of company size.

The necessity of experimental medicine is most acute when traditional endpoints are silent. Consider a hypothetical therapy targeting cellular energetics to reduce fibrosis. Such an agent may not reduce albuminuria in the short-term and might have no haemodynamic effect. In a typical Phase 2 trial relying on albuminuria reduction, this potentially disease-modifying drug would be deemed a failure. Only an experimental medicine study using biomarkers of inflammation, urinary proteomics, or imaging-based fibrosis assessment could reveal its therapeutic signal.

Experimental medicine studies in CKD can address critical questions that large trials cannot: haemodynamic effects through renal clearance studies using iohexol and PAH to distinguish haemodynamic from structural benefits; fibrosis modulation through kidney biopsies with advanced imaging and molecular profiling; metabolic reprogramming through stable isotope studies tracking metabolic flux in proximal tubule cells; and inflammatory modulation through urinary proteomics and single-cell RNA sequencing of urinary cells. These applications are characterised by rapid, reversible treatment effects that permit repeated within-patient measurement over clinically feasible timescales. By contrast, N-of-1 designs are poorly suited to interventions with prolonged washout periods or slowly accumulating effects, underscoring the need for careful alignment between study design and the therapeutic question.

## N-of-1 trials: deep insight from individual patients

Claude Bernard’s scepticism about statistics, that they yield conjecture rather than experimental certainty, finds its modern answer in N-of-1 trial methodology [16]. Population trials obscure individual responses through averaging; N-of-1 trials restore the patient as the fundamental unit of investigation, bringing systematic experimentation to the individual level. These studies, essentially randomised crossover trials in single patients, acknowledge a fundamental truth: the average patient does not exist. Every individual represents a unique combination of genetics, environment, comorbidities, and treatment responses. N-of-1 trials embrace this heterogeneity rather than trying to average it away.

The design is conceptually elegant [25] but methodologically demanding, requiring careful specification of treatment and washout periods, selection of sensitive and clinically meaningful outcomes, management of carryover effects, and appropriate statistical analysis. Its applicability is largely confined to conditions with stable baselines and reversible treatment effects. In nephrology, applications have included resistant hypertension management using home blood pressure monitoring, phosphate binder selection in dialysis patients, optimisation of dialysis circuit anticoagulation, and evaluation of treatments for uraemic pruritus, restless legs syndrome, or fatigue.

Beyond individual patient care, N-of-1 trials conducted in series generate valuable insights for drug development. They can reveal treatment effect heterogeneity, why some patients respond while others do not, informing precision medicine approaches [35]. Individual dose-response curves may vary substantially, and N-of-1 trials with frequent measurements can map temporal dynamics that population trials miss. By incorporating mechanistic measurements, urinary biomarkers, imaging, physiological assessments, they can link clinical responses to biological mechanisms.

Combining multiple N-of-1 trials creates distinctive analytical possibilities. Bayesian hierarchical models can pool individual patient data while retaining information about inter-individual variability, yielding both overall treatment estimates and identification of differentially responsive subgroups. Machine learning can detect patterns in this variability, potentially uncovering unexpected predictors of treatment response. Technology is making N-of-1 trials increasingly feasible: smartphone apps handle randomisation and data collection, wearable devices provide continuous physiological monitoring, and electronic health records capture laboratory results automatically.

## The path forward: integrating small and large

The future requires recognising that different study types answer different questions, all essential for therapeutic success. Development programmes should move iteratively between mechanistic studies and larger trials, with early experimental medicine identifying promising signals and biomarkers that inform Phase 2 design, and Phase 2 results triggering additional mechanistic studies to understand unexpected findings.

Consider a hypothetical illustration. A novel anti-inflammatory compound targeting IL-1β signalling advances to Phase 2 in diabetic kidney disease, demonstrating reduced albuminuria but also an unexpected 15–20% rise in serum creatinine during the first two weeks. Does this reflect nephrotoxicity or a beneficial haemodynamic effect, similar to the protective initial GFR dips seen with ACE inhibitors and SGLT2 inhibitors? Rather than proceeding directly to Phase 3 and risking premature termination, an experimental medicine study in a small number of participants could distinguish these mechanisms through serial inulin (or newer transdermal methods for measuring GFR [9]) and PAH clearance, filtration fraction assessment, and recognised urinary injury biomarkers (KIM-1, NGAL) *versus* podocyte stress markers (nephrin, podocalyxin). Completed within months at modest cost, such a study could reveal whether the creatinine rise reflects a potentially beneficial intraglomerular pressure reduction, providing mechanistic confidence for Phase 3. This illustrates the core principle: experimental medicine studies do not delay development but can de-risk it by transforming uncertainty into understanding.

However, reliance on non-validated biomarkers carries well-recognised risks. Progress through the FDA Biomarker Qualification Program has been slow, only 8 biomarkers have been qualified to date, and many proposals stall early [5]. We are not arguing that mechanistic biomarkers replace validated clinical endpoints. Rather, carefully characterised physiological measures can support critical development decisions such as dose selection, patient stratification and early detection of mechanism-based toxicity without substituting biological conjecture for clinical evidence.

### Cross-talk between data science and experimental medicine

Equally important is structured cross-talk between data scientists working with large datasets and experimentalists who can test the mechanistic hypotheses these analyses generate. Large-scale ‘omics and registry analyses increasingly identify associations, for example, between specific proteomic signatures and CKD progression, but associations are not mechanisms. Systematic frameworks for translating data-driven hypotheses into experimental medicine protocols would maximise the value of both approaches. Journal editors have a key role in this: rather than accepting database analyses that report only associations, editors could require authors to specify what experimental studies their findings suggest, creating a virtuous cycle between hypothesis generation and mechanistic testing.

### Regulatory and institutional reform

Regulatory agencies should formally recognise experimental medicine data in development programmes. The FDA’s recent willingness to accept eGFR slope as a surrogate endpoint represents progress [23, 37]; accepting validated mechanistic biomarkers and recognising N-of-1 trial evidence would be logical next steps. Strategic academic–industry partnerships should leverage their respective strengths: academic centres designing and conducting mechanistic studies, industry providing compounds, funding, and development expertise, and practice networks implementing N-of-1 trials to understand real-world effectiveness.

Reversing expertise erosion requires deliberate investment. Academic medical centres should establish dedicated experimental medicine fellowships combining comprehensive training in advanced techniques — microdialysis, hyperinsulinaemic–euglycaemic clamps, stable isotope tracing, continuous sampling methods, quantitative systems pharmacology — with apprenticeship in trial design and regulatory science. Dedicated research networks modelled on the NIHR Experimental Medicine network in the UK or Clinical and Translational Science Award (CTSA) centres in the US could provide shared infrastructure with specialised equipment, regulatory support, biostatistics expertise that individual institutions cannot easily maintain, particularly when these networks facilitate collaboration across international boundaries to overcome local academic silos.

Pharmaceutical companies should reform internal incentives. A project spending an additional year on experimental medicine that subsequently achieves regulatory approval may represent greater value than three projects rushed to Phase 3 where two fail. When senior leaders discuss mechanistic understanding alongside trial enrolment, and investor presentations highlight biological insights alongside timelines, prevailing conventions begin to change. Some progressive companies now require mechanistic evidence packages before advancing to Phase 3, making experimental medicine a gatekeeper rather than an optional detour.

## Conclusion: the wisdom of looking closely

Medicine advances through cycles of observation, hypothesis, and testing. The grand randomised controlled trial represents the testing phase, which is necessary for definitive proof but insufficient for understanding. Experimental medicine in all its forms, from traditional small cohort studies to Phase 1b trials to N-of-1 investigations, represents the observation and hypothesis phases, essential for generating insights that make definitive trials possible and interpretable.

The marginalisation of small mechanistic studies in favour of large pragmatic trials is a false economy. Even when therapies succeed in Phase 3, lack of mechanistic clarity can blunt their real-world impact. Recent analyses of CKD therapy uptake illustrate this problem: despite robust outcome data, SGLT2 inhibitors reach only 6–28% of eligible patients globally [18, 38]. Persistent gaps reflect more than drug access alone. Uncertainty about mechanism of action, patient selection, and early physiological effects can lead to clinical inertia and cautious institutional policies. Learning from successful implementation strategies using decision support tools, simplified initiation protocols, and specialist–primary care collaboration will be essential to ensure that mechanistic insights translate into changed practice.

The pioneers who advanced medicine through self-experimentation, Marshall with *H. pylori* and Forssmann with cardiac catheterisation, demonstrated that mechanistic inquiry, not just statistical power, drives discovery. While ethical protocols have appropriately replaced self-experimentation, we must not lose that spirit of curiosity and careful observation. In the end, experimental medicine reminds us that understanding disease is not just about proving what works but understanding why it works, in whom, and how we might make it work better. This understanding comes from biological insight, from deep observation, from scientific enquiry. As we develop new treatments for kidney disease and other complex conditions, sometimes the most important studies are the smallest ones. 

## Data Availability

No datasets were generated or analysed during the current study.
